# Cervical cancer elimination in Italy: Current scenario and future endeavors for a value based prevention

**DOI:** 10.3389/fpubh.2022.1010237

**Published:** 2022-11-30

**Authors:** Giovanna Elisa Calabrò, Maria Teresa Riccardi, Floriana D'Ambrosio, Carolina Castagna, Martina Sapienza, Rossella Millevolte, Andrea Pellacchia, Roberto Ricciardi, Rosa Pasqualina de Vincenzo, Chiara de Waure

**Affiliations:** ^1^Section of Hygiene, University Department of Life Sciences and Public Health, Università Cattolica del Sacro Cuore, Rome, Italy; ^2^VIHTALI Value in Health Technology and Academy for Leadership and Innovation, Spin Off of Università Cattolica del Sacro Cuore, Rome, Italy; ^3^Department of Medicine and Surgery, University of Perugia, Perugia, Italy; ^4^Gynecologic Oncology Unit, Fondazione Policlinico Universitario A. Gemelli, IRCCS, Dipartimento Scienze della Salute della Donna, del Bambino e di Sanità Pubblica, Rome, Italy; ^5^University Department of Life Sciences and Public Health, Università Cattolica del Sacro Cuore, Rome, Italy

**Keywords:** cervical cancer, value-based prevention, vaccination, screening, indicators

## Abstract

**Background:**

Cervical Cancer (CC) is a vaccine-preventable disease, and it is treatable if diagnosed early and managed properly. However, it is the fourth most common cancer in women worldwide with about 604,127 cases and 341,831 deaths in 2020. In Italy, it represents the fifth most common cancer in women under 50 years of age with about 2,400 new cases in 2020. The CC elimination is today a global public health goal published by the World Health Organization (WHO) in 2020 and a commitment of the European Union that has included it in Europe's Beating Cancer Plan. Therefore, urgent action is needed, at international and national level, to implement value-based interventions regarding vaccination, screening and timely management of the disease. Our study aims to describe the state of the art of Human Papilloma Virus (HPV) prevention in Italy and to get a consensus on indicators for monitoring the progress toward CC elimination at national level.

**Methods:**

The study envisaged the following activities: research and synthesis of the evidence on strategies and actions for CC elimination at regional Italian level; identification of indicators to monitor such strategies/actions; organization of a multi-stakeholder consensus to reach the agreement on main indicators to be used in Italy.

**Results:**

As for HPV vaccination coverage, the last Italian available data (December 31st, 2020) showed that it was way below the target (95%) with full cycle vaccination coverage ranging from 6 to 61.7% in female adolescents and from 5.4 to 55.4% in male adolescents (2008 birth cohorts). The coverage rate of CC screening is variable with a range of 61.7–89.6%. Furthermore, coverage rates due to organized screening programs (excluding out-of-pocket screening) shows a range from 20.7 to 71.8%. The mapping of the Italian Regions highlighted an important regional heterogeneity in respect to organizational/operational issue of HPV vaccination and CC screening. Indicators for monitoring CC elimination strategies have been drawn from the Australian experience and distinguished by disease outcomes, vaccination coverage, screening participation and treatment uptake. The highest consensus was reached for the following indicators: CC incidence; detection of high-grade cervical disease; CC mortality; full cycle vaccination coverage; screening participation; high-grade cervical disease treatment rates; CC treatment rates.

**Conclusions:**

The assessment of the current status of CC elimination as overarching goal beyond the achievement of vaccine, screening and treatment targets represents the first step for the identification of interventions to be implemented to accelerate the path toward CC elimination. Based on this and following the WHO call, a value-based approach is proposed to untangle the full benefit of HPV-related cancers elimination strategies and identify priority and best practices.

## Introduction

Human Papilloma Virus (HPV) has been recognized as a carcinogenic agent since 1995 (1). Cervical cancer (CC) accounts for around 80% of all HPV-related cancers (2) and HPV types 16 and 18 are responsible for 72% of all HPV-related cancers whereas HPV31, 33, 45, 52, and 58 account for an extra 17% (3). These HPV types are categorized as oncogenic high-risk (HR) (4).

More than 95% of CC is due to the HPV (5). Most sexually active women and men are infected in their lifetime, and some may be repetitively infected. More than 90% of the infected cases resolve spontaneously, with viral clearance, within two years; however, the persistence of HR-HPV infection can lead to dysplasia and an increased risk of developing cancer (6).

About 604,127 new CC cases are diagnosed annually worldwide with about 342,000 deaths each year (estimations for 2020); this cancer represents the 4th leading cause of woman cancer and the 2nd most common cancer in women aged 15–44 years in the world (4). Furthermore, CC represents the 9th most frequent cancer among European women with more than 58,000 new cases and almost 26,000 deaths each year (4). Epidemiological data vary deeply across Europe also because of differences in prevention policies (7). In Italy, the age standardized incidence rate of CC is 6.9 per 100,000 women (4) with 2,400 new cases in 2020 (8).

CC is a preventable disease, through HPV vaccination and screening, and it is treatable if diagnosed early and managed properly (5). However, the burden of CC is still relevant worldwide. In this light, a Global strategy toward eliminating CC as a public health problem, has been adopted by the World Health Assembly in 2020 (9). This strategy includes a comprehensive approach to CC prevention and control, and proposes lifelong actions through primary, secondary and tertiary prevention interventions. In particular, the strategy of World Health Organization (WHO) proposes a threshold of 4 per 100,000 women-years for CC elimination, and the 90-70-90 actions targets to be met by 2030, namely 90% of girls fully vaccinated with HPV vaccine by age 15 years, 70% of women screened with a high-performance test by 35 years of age and again by 45 years of age, and 90% of women with a CC receiving treatment (90% of women with pre-cancer treated, and 90% of women with invasive cancer managed) (9). Furthermore, according to the WHO, CC prevention should involve a multidisciplinary approach, including community education, social mobilization, vaccination, screening, treatment and palliative care (5, 9).

Following the WHO call, in February 2021, the European Commission (EC) published the Europe's Beating Cancer Plan with the aim of promoting a common fight against the cancer in all European Union (EU) Member States. One of the proposed initiatives concerns precisely the elimination of CC and other HPV-related cancers through the achievement of the 90-90-90 targets by 2030 (10).

In Italy, the current National Immunization Plan (NIP) 2017–2019 provides free HPV vaccination in girls and boys aged 12 years of age and sets a 95% target vaccination coverage (11). Additionally, the NIP recommends HPV vaccination for Men who have Sex with Men (MSM) and women 25 years old, also using the opportunity of the call to the first screening for cervical cancer. The NIP also recommends vaccination according to the guidelines of the Regions (co-payment scheme) for all women (11). Instead, as regards CC screening, the National Prevention Plan 2014–2018 (12) provided that all Italian regions by 2018 passed from the Pap test to HPV-DNA as the primary test for women aged 30–35. In the National Plan 2020–2025 (13) it is planned to continue in completion of this transition in all regions.

In a current context characterized by increasing economic pressure, health systems worldwide face challenges related to the need to ensure access to high quality healthcare for all citizens. Therefore, evidence-based tools to support a value-based decision-making process are needed also in the prevention field (14, 15). Understanding of the value should be shared by all health actors and be geared toward the goal of maximizing social wellbeing (16). In fact, we are moving from the concept of a value-based health care to the concept of a value-based health system as it is the whole health system that contributes to societal wellbeing (16), thanks also to health promotion and prevention interventions. However, this cannot disregard a deep knowledge of the current scenario. From this perspective, considering the autonomy granted to Italian regions in respect to the development of health strategies, this work was aimed at mapping vaccination and screening policies and strategies in the 20 Italian regions and identifying, through the consultation of a board of Italian experts, the indicators for monitoring the progress toward CC elimination at national level.

## Materials and methods

A two-pronged method was used to conduct the study. First, a search of documents and data on HPV vaccination and CC screening policies and strategies in the 20 Italian regions was conducted from June 2021 to March 2022. For this purpose, institutional websites—such as those of the Ministry of Health (www.salute.gov.it), of the National Institute of Health (www.epicentro.iss.it) and of the National screening observatory (www.osservatorionazionalescreening.it) -, regional websites, and the website of the Italian Group for Cervical Cancer Screening (GISCi) (www.gisci.it), were queried using the following search terms: HPV elimination strategies, HPV vaccination uptake and cervical screening. The mapping process was performed by six researchers independently. Then, the items reported in Table 1 were collected in an excel-sheet for each Italian region.

**Table 1:** Items on HPV vaccination and cervical screening collected in the mapping process of our study, for each Italian region.

**Items on HPV vaccination**	**Items on cervical screening**
Full cycle vaccination coverage in adolescents (females and males)	Cervical screening coverage (total coverage and organized screening coverage)
Vaccination reimbursement policies (free of charge/co-payment) for different targets such as: - adolescents, - people with HIV infections, - Men who have Sex with Men (MSM), - people with previous diagnosis of HPV-related lesions. Presence/absence of a regional coordination on vaccination	Screening extension and adherence with different HPV detection methods (HPV-DNA test/Pap smear testing) Target age for screening with HPV-DNA test Presence/absence of a specific strategy for women previously vaccinated for HPV Presence/absence of a regional coordination on screening Presence/absence of a regional diagnostic-therapeutic-care pathway (DTCP) for CC management.

The latest data available on HPV vaccination and screening coverage in Italy refer to the year 2020 while the latest Italian data on cervical screening extension and adherence are those of 2018.

Each Italian region excel-sheet was subjected to the double check of two researchers and then double-checked further by two senior researchers.

Second, a multidisciplinary and multi-stakeholder board of experts has been established to evaluate and validate collected information and data and to achieve a consensus over indicators to monitor the progress toward CC elimination at national level.

The board was made up of 17 experts selected among health care professionals with relevant knowledge and experience in HPV-related diseases prevention and management: four members of the Italian scientific society of hygiene, preventive medicine and public health experts in the vaccination field; two members of GISCi experts in cancer screening; two gynecologists, an oncologist, a referent of the Italian cancer registry, an andrologist, a radiotherapist, three pediatricians (two from the hospital setting and one from the territorial setting), an otolaryngologist and a general practitioner, all referents of their respective scientific societies.

The board was involved in two virtual meetings and was requested to answer an online survey launched through Google Platform in between the two meetings. In the first virtual meeting, in December 2021, the research working group shared collected information and data and introduced the indicators to monitor CC elimination in Italy. Regarding indicators, the Australian report on progress toward the elimination of CC was considered (17). Australia is a world leader in CC prevention and control, having achieved a halving of incidence and mortality through the cytology-based National Cervical Screening Program first implemented in 1991; and the world's first national HPV vaccination program in 2007, which resulted in a significant reduction in rates of HPV infection and precancerous cervical lesions. With the transition from cytology to HPV based screening in December 2017 and the introduction of the nonavalent HPV vaccine in 2018, Australia is expected to be the first country to reach the WHO definition of eliminating CC as a public health problem by 2030. Australia is also a world leader in research and surveillance documenting the impact of CC control programs. In fact, since 2018, Australian public health and clinical researchers have been collaborating in the Center of Research Excellence in Cervical Cancer Control. Additionally, Australia was the first country to produce a comprehensive report on Australia's progress toward CC elimination, proposing 11 key indicators to monitor progress toward the achievement of WHO objectives (17).

For these reasons, the 11 Australian indicators grouped into four components (disease outcomes, vaccine coverage, screening participation, treatment) were proposed to the group of Italian experts in order to evaluate their applicability and usefulness in Italy.

The online survey was aimed at collecting experts' positions in respect to the utility of the 11 indicators (binary response: yes/no). The survey was live for four weeks to allow all experts to take part and a reminder was sent one week prior to the deadline to ensure maximum participation.

In the second virtual meeting, results of the survey were shared and a final agreement was reached though a plenary discussion led by three experts, scientific managers of the project (two experts in public health and a gynecologist).

These activities were completed on March 9, 2022.

## Results

The main results of our study are reported in the following sections: HPV vaccination in Italian regions, screening for CC in Italian regions, and results from the experts' consultation.

The results of the mapping of policies and strategies in Italian regions are summarized in Tables 2, 3.

**Table 2:** Summary of information and data on HPV vaccination policies and strategies in the Italian regions.

	**Abruzzo**	**Basilicata**	**Calabria**	**Campania**	**Emilia Romagna**	**Friuli Venezia Giulia**	**Lazio**	**Liguria**	**Lombardia**	**Marche**	**Molise**	**Trentino Alto Adige-AP Bolzano**	**Trentino-Alto Adige–AP Trento**	**Piemonte**	**Puglia**	**Sardegna**	**Sicilia**	**Toscana**	**Umbria**	**Valle d'Aosta**	**Veneto**
**HPV—Vaccination**																					
Vaccination coverage—girls (2008 cohort) (%)	29.8	43.6	40.9	24.8	51.1	9	19.1	46.2	17.8	29	34.8	13.9	61.7	49	44.8	15	22.6	53.4	53.9	6	17.9
Vaccination coverage—boys (2008 cohort) (%)	18.5	38.2	31.2	12.8	46.9	8	9.6	37	16.6	22.5	29.5	10.4	55.4	43.5	39.1	12.3	14.9	40.5	46.9	5.4	16.4
Free vaccination in the female population without limits of age									X					X							
Free of charge vaccination of people with HIV infections					X	X	X	X	X	X		X	X					X			X
Free of charge vaccination of people at risk and MSM		X				X	X		X		X	X	X	X	X			X			X
Free of charge		X		X	X	X	X	X	X	X	X	X	X	X	X	X	X	X	X		X
vaccination of people with previous diagnosis of HPV-related lesions		M/W									M/W						M/W				
Co-payment for all	X		X		X	X	X			X		X	X	X	X				X		X
other groups	M/W		M/W		M/W	M/W	M/W			W		M/W	M/W	M/W	M/W				M/W		M/W
Regional coordination		X			X			X		X				X				X			X

MSM, Men who have Sex with Men; M/W, for men and women; AP, autonomous provinces.

**Table 3:** Summary of information and data on cervical screening policies and strategies in the Italian regions.

	**Abruzzo**	**Basilicata**	**Calabria**	**Campania**	**Emilia Romagna**	**Friuli Venezia Giulia**	**Lazio**	**Liguria**	**Lombardia**	**Marche**	**Molise**	**Trentino Alto Adige-AP Bolzano**	**Trentino-Alto Adige-AP Trento**	**Piemonte**	**Puglia**	**Sardegna**	**Sicilia**	**Toscana**	**Umbria**	**Valle d'Aosta**	**Veneto**
**Cervical screening**																					
Coverage, 2020 (%)	75.7	73.6	61.7	64.9	89.3	89.5	85.9	86.5	83.4*	84.6	64.9	89.6	85.1	84.5	75.4	73.3	69.5	88.2	87.3	64.9	88.4
Coverage of organized screening, 2020 (%)	45.6	45.6	33.6	20.7	68.5	66.5	39	41.1	31.2*	56.1	27.6	56	57.7	63.7	33.5	58	46.7	71.8	66.5	27.6	60.7
Extension of HPV-DNA test, 2018 (%)	72	70.1	2.1	2.4	100	0	69	9.6	1.7	0	53.4	13.5	100	100	0	0	10.6	81.5	75.6	88.2	100
Adherence to HPV-DNA test, 2018 (%)	56.7	62.7	28.7	87.6	61.1	0	29.3	82.8	46.7	0	78.3	33.6	66.8	43.9	0	0	19.8	53.4	61.9	71.8	63.5
Extension of pap- test, 2018 (%)	9.5	12.7	23.5	55.7	29.6	80.3	55.6	83.9	22.9	86.6	83.9	94.8	38.9	15.6	84.2	80.7	82.6	38.6	8.3	54.4	10.1
Adherence to pap-smear test, 2018 (%)	90.3	59	31.7	23.8	58.2	64.5	27.3	29.8	49.9	42.4	5.1	30.1	53	44.5	33.2	41.8	21.6	51	68.5	64.3	59
Regional coordination	X	X	X	-	X	X	X	X	X	X	X	X	X	X	X	X	X	X	X	X	X
Target	>30	>35	>30	>35	>30	>35	>30	>30	>35	>30	>35	>30	>30	>30	≥25	>30	>35	>35	>30	>30	>30
population of															(from						
HPV-DNA															September						
test (years															2022)						
old)																					
Regional DTCP		X		X	X		X			X					X			X	X		
Specific strategy for previous vaccinated women																					X

DTCP, Diagnostic-therapeutic-care pathway; AP, autonomous provinces; *Data refer to the year 2019.

### HPV vaccination in Italian regions

Despite the efforts to reach the goal of 95%, the last Italian available data (December 31st, 2020) showed that vaccination coverage was way below the target with full cycle vaccination coverage ranging from 6 to 61.7% in female adolescents (2008 birth cohort), and from 5.4 to 55.4% in male adolescents (2008 birth cohort). Table 2 shows an important variability among the Italian regions and the two autonomous provinces (A.P.) both for vaccination coverage and for HPV vaccination policies and strategies. For example, as of March 2022 Piemonte and Lombardia regions provide girls who were included in the target population with lifetime free of charge access to vaccination. Free of charge vaccination is also offered to people belonging to at-risk groups, as follows: people with diagnosis of HIV infection in eight regions and in two A.P. (47.6%) and MSM in 9 regions and in two A.P. (52.4%). Regarding people with HPV-related lesions, 16 regions and two A.P. (85.7%) offer free of charge vaccination to women and three out of them also offer it to men. However, it should be noted that from March to today the offer free of charge vaccination to women with HPV-lesions has also been extended to the other Italian regions. Co-payment with no age limit is provided in 57% of regions (10 regions and 2 A.P), but one of them (Marche) offers this service only to women. The presence of coordination at a regional level is evidenced in seven regions (33.3%).

### CC screening in Italian regions

The screening coverage data refer to 2020, except for Lombardy whose available data are updated to 2019. The coverage rate of CC screening is variable (Table 3) with a range of 61.7–89.6%. Furthermore, coverage rates due to organized screening programs (excluding out-of-pocket screening) shows a range from 20.7 to 71.8%.

At the time of our analysis, the transition from the Pap test to HPV-DNA as the primary test for women aged 30-35 proposed by the National Prevention Plan 2020–2025 (13) was still ongoing in one region (Puglia). Furthermore, data for the calculation of the indicators of screening extension and adherence refer to 2018, when the programs with HPV-DNA were in progress in all regions except four (Friuli Venezia Giulia, Marche, Puglia and Sardinia). Indicators were calculated for both HPV-DNA test and Pap smear. As regards HPV-DNA test the extension ranges from 0 in the Friuli Venezia Giulia, Marche, Puglia and Sardinia regions to 100% in the Emilia-Romagna, AP of Trento, Piemonte and Veneto; the adherence ranges from 19.8% in Sicily to 87.6% in Campania. As regards Pap smear test, the extension ranges from 8.3% in the Umbria region to 94.8% in the AP of Bolzano; the adherence ranges from 5.1 in the Molise region to 90.3% in the Abruzzo region.

As of January 2022, target populations for HPV-DNA test differ across the regions. In 13 (65%) regions, HPV-DNA testing is offered to women older than 30 years of age, whereas in seven (35%) the target population is represented by women older than 35 years of age. Recently, the Puglia region disclosed that it will perform the HPV-DNA test starting from the age of 25 (start of the program from September 2022).

At the time of data analysis, the presence of a regional coordination for the screening is evidenced in all regions and A.P. except for Campania region.

The presence of a regional DTCP specific for CC management was available in eight regions (38.1%).

Eventually, regarding the rescheduling of the screening for women vaccinated with a complete cycle, within the National Prevention Plan 2020–2025 (13) is given explicit mandate to the Regions to draw up a specific evidence-based strategy, and, currently, the first Region that has implemented this recommendation is Veneto.

### Results from the experts' consultation

The response rate to the online survey was 100%. The results regarding the agreement on the utility of the 11 indicators drawn from the Australian experience are reported in Table 4. Overall, the majority of experts agreed on the utility of all the indicators. Nonetheless, in respect to diseases outcome, CC incidence and detection of high-grade cervical disease in the screened women have reached unanimous consent. In respect to vaccination, full cycle vaccination coverage was assigned most importance. In respect to screening and treatment, screening participation and treatment rates of high-grade cervical disease and CC reached the highest consensus. Nevertheless, from the plenary discussion it emerged that the monitoring of adherence to screening at 35 and 45 years of age is relevant and possible at national/regional level.

**Table 4:** Evaluation of the Italian experts regarding the 11 indicators to be used to monitor the interventions to be implemented for the CC elimination in Italy.

**Indicators**	**Do you agree with the evaluation of the following indicators to monitor the progress toward cervical cancer in Italy?**	**Yes *N* (%)**	**No *N*(%)**
**Disease outcomes**	CC incidence	17 (100%)	0 (0%)
	Detection of high-grade cervical disease	17 (100%)	0 (0%)
	CC mortality	16 (94.1%)	1 (5.9%)
	Prevalence of HPV infection	15 (88.2%)	2 (11.8%)
**Vaccine coverage**	HPV vaccine initiation by age 15	13 (76.5%)	4 (23.5%)
	HPV vaccine completion by age 15	16 (94.1%)	1 (5.9%)
**Screening participation**	Screening participation	15 (88.2%)	2 (11.8%)
	Screening participation by age 35 and 45 year	12 (70.6%)	5 (29.4%)
**Treatment uptake**	Colposcopy attendance	14 (82.4%)	3 (17.6%)
	High-grade cervical disease treatment rates	15 (88.2%)	2 (11.8%)
	Cervical cancer treatment rates	15 (88.2%)	2 (11%)

## Discussion

This paper reported the status of vaccination and screening policies and strategies in Italy highlighting two main important issues: the first one is that Italy must still work to achieve the targets of the WHO Global Strategy whereas the second one is that Italian regions show an undue variability that might slow down the achievement of the targets.

Considering the actions proposed by the WHO strategy and in particular the goal of vaccinating 90% of girls with HPV vaccine by age 15 years, our data shows that if the provision of vaccination to the target group is satisfied in all Italian regions, coverages are still not optimal. Furthermore, referring to the other NIP indications, namely vaccination in MSM and in women aged 25 years old, only slightly more than half of the regions offer the HPV vaccination to the MSM, and still not all regions actively offer vaccination at the first screening.

However, it is important to point out that some Italian regions have extended the vaccination to other targets at risk, such as, for example, women treated for HPV-related lesions.

On the contrary, with respect to the possibility to access vaccination in co-payment all regions are aligned, with someone even providing for the extension to the male population. In addition, worthy of note is to report that, at the time of our analysis, two Italian regions (Lombardia and Piemonte) reserve free of charge vaccination to life for women who have returned to the primary target, a strategy that can facilitate the improvement of vaccination coverage.

Nevertheless, as vaccination coverage in Italy is still very far from the 90% target set by WHO it is necessary to implement targeted actions aimed at implementing health education, as also proposed in the WHO strategy (9). Furthermore, combining education, information, and communication activities with other kinds of interventions could led to more effective and lasting results. In fact, multicomponent strategies are shown to achieve the best results (18).

Indeed, the Italian national health system should work on the integration of different approaches, including personalized reminders, information and educational activities aimed at increasing adolescents', parents' and healthcare professionals' (HCPs) awareness and knowledge about HPV infection and vaccination, training programs for HCPs on communication strategies with parents and adolescents, and facilitated access to vaccination also including vaccination programs in schools (18) as done in Australia since 2007 (17).

Australia, thanks to the primary and secondary prevention strategies implemented (19, 20), is expected to be the first country to reach the WHO definition of eliminating CC as a public health problem by 2030. In fact, in 2011–2015, the annual incidence of CC in Australia was 6.3 cases per 100,000 women (17) and it has been projected to decrease below 4 new cases per 100,000 women by 2030 (17).

In respect to screening, in Italy, the coverage is largely variable across regions and A.P. Furthermore, considering that the objective set by the WHO refers to the HPV-DNA test, it must certainly be highlighted that yet not all the Italian regions have completed the process of implementation of the HPV-DNA test within screening programs. Moreover, also in the regions where the HPV-DNA test is offered, an extension of 100% is not always achieved. It should be also noted that adherence to the screening with HPV-DNA testing is still extremely variable among different Italian regions. It follows that even in respect to screening there is still a lot to work. Surely greater regional coordination action, currently present in almost all regional realities, would allow for a more homogeneous offer.

Despite the effectiveness and cost-effectiveness of prevention interventions, investment in disease prevention remains low in many countries (21). Among the barriers, there are the unwillingness to invest in actions that generally generate positive benefits in the long-term horizon and the difficulty of different actors to immediately enjoy the health benefits obtained from prevention (16). Therefore, in order to remove these barriers and to improve the citizens' health and the health systems value, especially in priority areas for public health such as that of the control of HPV-related cancers, actions should be taken following the concept of value proposed by Expert Panel on Effective Ways of Investing in Health (EXPH) of the EC in 2019. The proposed concept is built on four value-pillars: appropriate care to achieve patients' personal goals (personal value), achievement of best possible outcomes with available resources (technical value), equitable resource distribution across all patient groups (allocative value) and contribution of healthcare to social participation and connectedness (societal value)” (22). This approach is also in line with the perspective of a value-based health system proposed by the WHO and the European Observatory on Health Systems and Policies (16). According to these international institutions, the main objective of health systems is to maximize social wellbeing, understood as the value created by the system as a whole, including health promotion and disease prevention (16). In particular, as stated by the EXPH, the guiding principles are access, equity, quality, performance, efficiency and productivity (optimization and distribution of resources) (22).

According to this value-based perspective, the involvement of all stakeholders—governments, scientists, healthcare professionals, patients and citizens, providers and industries—is the key to implement high-value health care (22). Similarly, an appropriate governance is necessary (16). Greer and colleagues (23) present a five-dimensional framework for designing and assessing governance of health systems, defined TAPIC (Transparency, Accountability, Participation, Integrity, and Policy capacity). This framework also underlines the need to identify useful indicators to measure health improvements associated with value-based interventions (16).

The lack of nationwide data on the whole HPV-related diseases epidemiology and treatment indicators could undermine the assessment of the quality and the performance of the health system. In fact, a fundamental action for a proper governance of healthcare and health systems as a whole is the identification and the routine use of indicators, as done in Australia for monitoring CC elimination. Our survey with the experts revealed the utility and applicability of the Australian indicators also in Italy, even though some critical issues have been pinpointed in respect to the availability and access to data in particular in respect to disease outcomes and treatment. In this respect, the active and informed involvement of all relevant stakeholders (14) will play a fundamental role in both making the constant evaluation possible and ultimately achieving the goal of eliminating the CC and controlling all other HPV-related diseases.

In September 2022, the document “Roadmap to accelerate the elimination of cervical cancer as a public health problem in the WHO European Region 2022–2030” was also published with the aim to implement the Global strategy to accelerate the elimination of CC as a public health problem in the European Region (24). This document emphasizes that robust surveillance and health information systems are critical for monitoring and evaluating the impact of the proposed roadmap. Furthermore, it is proposed that Member States should develop or update their national action plans, outlining clear strategies and mechanisms to achieve the targets and goals outlined in the regional roadmap; and that Member States should develop costed comprehensive national action plans with priority actions and a monitoring, evaluation and accountability framework, with active engagement from national, regional and global stakeholders. As with Australia, the European roadmap, in line with the global strategy, will need to include metrics to monitor regional progress toward the 2030 global goals and to assess progress on the path to CC elimination. In addition, an interim report on the progress made in the European Region for the CC elimination is planned and it will be presented to the WHO Regional Committee for Europe in 2026 (24).

Our study is in line with the guiding principles of the WHO European Region roadmap, in particular with regard to the definition of indicators for monitoring CC elimination strategies.

The importance of indicators was also emphasized in the Europe's Beating Cancer Plan. In fact, the European Cancer Inequalities Registry (25) was created to provide reliable data on cancer prevention and care to identify trends, disparities and inequalities between Member States and European regions. This registry proposes the following indicators for monitoring data on CC elimination: death rate per 100,000 women due to CC, the percentage of girls (aged 15 years old) who received a recommended dose of HPV vaccine, the percentage of women aged 20–69 who reported to have never had cervical smear test.

According to data reported by the European register, Italy is with France and Bulgaria among the European countries with the lowest HPV vaccination coverage. On the other hand, for the screening indicator, Italy in line with the European average (25).

The Australian experience and our study emphasized that attention should be paid also to other indicators to comprehensively monitor the attainment of the targets proposed by the Global strategy for the CC elimination.

Our study has some limitations. First, collected data are not updated to the last year. Furthermore, a selection bias could not be completed ruled out even though the mapping process was performed by six researchers independently and based on specific criteria. Eventually, the heterogeneity of data limits the possibility to further elaborate on information and issue more definite findings. Nevertheless, in our opinion, this first Italian regional mapping on prevention interventions for the CC control could help bringing forward the assessment and the appraisal of the health policies in this field from the point of view of both academic research and supranational, national and local decision-makers.

## Conclusions

The mapping of the Italian Regions highlighted that HPV vaccination coverage and cervical screening coverage are still too low to achieve CC elimination by 2030; furthermore, an important regional heterogeneity was shown in respect to primary and secondary prevention policies, strategies and implementation status. Therefore, our study highlighted room for improvement regarding several issues, as the highlighted heterogeneity imply great differences in terms of access and equity.

The assessment of the status of achievement of vaccination, screening and treatment goals and the identification and constant assessment of specific indicators for monitoring the progress toward CC elimination are fundamental actions to be able to respond to the WHO call. To achieve the goals proposed by the global strategy for the CC elimination, all available means must be used, also in Italy, focusing on a comprehensive approach in favor of value-based effective interventions of prevention and best practices to be implemented at regional level.

## Data availability statement

The original contributions presented in the study are included in the article/supplementary material, further inquiries can be directed to the corresponding author.

## Author contributions

Conceptualization and methodology, project administration, and funding acquisition: GEC. Definition of the search strategy, supervision, and data review: GEC and CdW. Search of documents on HPV vaccination and CC screening policies and strategies in Italian regions: MTR, FD'A, CC, MS, RM, and AP. Data analysis: MTR and FD'A. Writing—original draft preparation: MTR, FD'A, GEC, and CdW. Writing—review and editing: GEC, RR, RPdV, and CdW. All authors have read and agreed to the published version of the manuscript.

## Funding

This study was financed by an unconditional grant from MSD Italia S.r.l. The sponsor had no role in conducting or designing the study, collecting, analyzing, interpreting the data, and the writing the manuscript. Universitá Cattolica del Sacro Cuore contributed to the funding for this publication with funds from UCSC-Line D.1 2022.

## Conflict of interest

All the authors worked as consultants of VIHTALI (Value in Health Technology and Academy for Leadership & Innovation), Spin-Off of Università Cattolica del Sacro Cuore (Rome, Italy), and which received funds from MSD Italia S.r.l. The sponsor had no role in conducting or designing the study.

## Publisher's note

All claims expressed in this article are solely those of the authors and do not necessarily represent those of their affiliated organizations, or those of the publisher, the editors and the reviewers. Any product that may be evaluated in this article, or claim that may be made by its manufacturer, is not guaranteed or endorsed by the publisher.
